# Quantum Mechanics/Molecular Mechanics Studies on the Relative Reactivities of Compound I and II in Cytochrome P450 Enzymes

**DOI:** 10.3390/ijms19071974

**Published:** 2018-07-06

**Authors:** Verònica Postils, Maud Saint-André, Amy Timmins, Xiao-Xi Li, Yong Wang, Josep M. Luis, Miquel Solà, Sam P. de Visser

**Affiliations:** 1 Institut de Química Computacional i Catàlisi (IQCC) and Departament de Química, Universitat de Girona, Maria Aurèlia Capmany i Farnés, 69, 17003 Girona, Catalonia, Spain; vpostils@gmail.com (V.P.); josepm.luis@udg.edu (J.M.L.); 2 Manchester Institute of Biotechnology, School of Chemical Engineering and Analytical Science, The University of Manchester, 131 Princess Street, Manchester M1 7DN, UK; maudstandre@gmail.com (M.S.-A.); Amy.timmins@postgrad.manchester.ac.uk (A.T.); 3 State Key Laboratory for Oxo Synthesis and Selective Oxidation, Suzhou Research Institute of LICP, Lanzhou Institute of Chemical Physics (LICP), Chinese Academy of Sciences, Lanzhou 730000, China; xiaoxi870808@gmail.com (X.-X.L.); wangyong@licp.cas.cn (Y.W.)

**Keywords:** enzyme mechanism, enzyme catalysis, heme, iron, hydrogen atom abstraction, density functional theory, QM/MM, inorganic reaction mechanism

## Abstract

The cytochromes P450 are drug metabolizing enzymes in the body that typically react with substrates through a monoxygenation reaction. During the catalytic cycle two reduction and protonation steps generate a high-valent iron (IV)-oxo heme cation radical species called Compound I. However, with sufficient reduction equivalents present, the catalytic cycle should be able to continue to the reduced species of Compound I, called Compound II, rather than a reaction of Compound I with substrate. In particular, since electron transfer is usually on faster timescales than atom transfer, we considered this process feasible and decided to investigate the reaction computationally. In this work we present a computational study using density functional theory methods on active site model complexes alongside quantum mechanics/molecular mechanics calculations on full enzyme structures of cytochrome P450 enzymes. Specifically, we focus on the relative reactivity of Compound I and II with a model substrate for O–H bond activation. We show that generally the barrier heights for hydrogen atom abstraction are higher in energy for Compound II than Compound I for O–H bond activation. Nevertheless, for the activation of such bonds, Compound II should still be an active oxidant under enzymatic conditions. As such, our computational modelling predicts that under high-reduction environments the cytochromes P450 can react with substrates via Compound II but the rates will be much slower.

## 1. Introduction

The cytochromes P450 are important heme monoxygenases in the body and particularly prevalent in the liver, where they catalyze the biosynthesis of several hormones including estrogen and also take part in the biodegradation and metabolism of toxic compounds [1,2,3,4,5,6,7,8,9]. In general, the P450s bind molecular oxygen on an iron(III)-heme group and transfer one of its oxygen atoms to a substrate through either an aliphatic C–H hydroxylation, aromatic C–H hydroxylation, C=C epoxidation or sulfoxidation [10,11,12,13]. The second oxygen atom originating from O_2_ is typically reduced to a water molecule. Due to its substrate versatility and influence on drug metabolism in the human body, the P450s have been the topic of many research studies. However, there are still many questions surrounding the catalytic mechanism of these enzymes.

**Figure 1:**
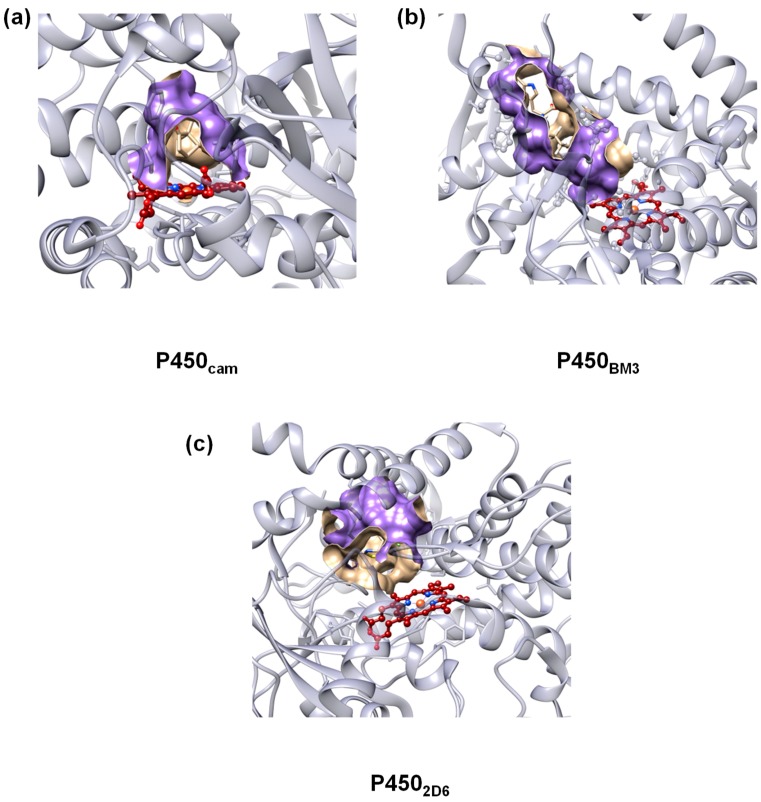
Active site structures of (**a**) P450_cam_; (**b**) P450_BM3_ and (**c**) P450_2D6_ with the substrate binding pocket shape highlighted in purple. The protein backbone is in light blue and the heme in red.

To accommodate for the substrate versatility, the P450s have a substrate binding pocket with a size that is dependent on the P450 isozyme. Thus, P450_cam_ [14,15,16], which is a camphor hydroxylating P450 isozyme where atom C^5^ of the substrate is regioselectively hydroxylated, has a binding pocket that tightly binds camphor in a specific orientation that enables this selective reaction. To illustrate the size and shape of the substrate binding pocket in P450 isozymes, we show in Figure 1 the crystal structure extracts of protein databank (pdb) files for P450_cam_ (2CPP pdb [17]), P450_BM3_ (3WSP pdb [18]) and P450_2D6_ (5TFT pdb [19]). P450_BM3_ binds long chain fatty acids and hydroxylates these at one of the carbon atoms in the terminus, whereas P450_2D6_ has a more spherical substrate-binding pocket as it is involved in the biodegradation of xenobiotics in the liver. These P450 isozymes, therefore, have distinct and different substrate binding pockets to accommodate for the substrate-types they activate.

**Figure 2:**
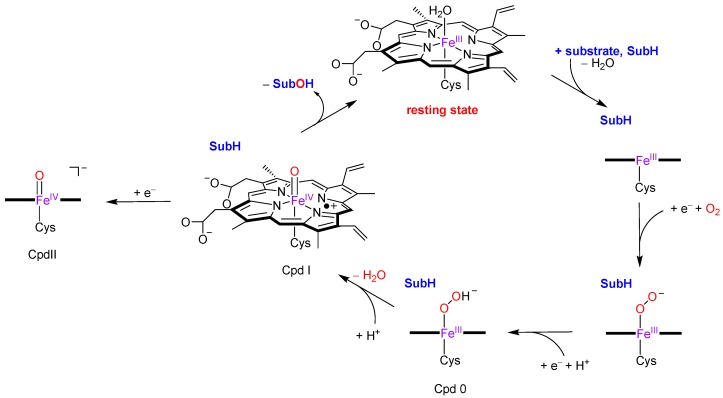
Consensus catalytic cycle via Cpd I and possible alternative via Cpd II.

The consensus catalytic cycle of P450 enzymes is schematically depicted in Figure 2 [20,21,22]. The cycle starts from an iron (III)-water bound heme structure, the resting state, that upon substrate (SubH) binding in the substrate binding pocket releases the water molecule. Then, iron (III) (heme)—SubH is reduced by the reduction partner and binds molecular oxygen as an iron (III)-superoxo, that is reduced again and protonated to form the iron (III)-hydroperoxo (heme) intermediate called Compound 0 (Cpd 0). The latter was trapped at low temperature and characterized with electron paramagnetic resonance studies [23]. A final protonation of Cpd 0 forms the most likely active species, namely Compound I (Cpd I), which was trapped and characterized by Rittle and Green [24] with UV-Vis absorption, electron paramagnetic resonance and Mössbauer spectroscopy. Cpd I reacts with aliphatic substrates through hydrogen atom abstraction followed by OH rebound to form alcohol product complexes (SubOH) [25,26,27]. After binding a water molecule, the intermediate returns to the resting state and is ready for the next catalytic cycle.

Despite the fact that Cpd I has been detected and characterized for one specific P450 isozyme, there are still on-going discussions on what the actual reactive species in P450 enzymes is and as such, a multiple oxidant hypothesis has been proposed [28]. These studies initially pointed to Cpd 0 as a possible “second oxidant”, but a series of computational studies by model complexes [29,30] as well as using quantum mechanics/molecular mechanics (QM/MM) calculations [31] ruled this hypothesis out. Subsequent experimental studies on biomimetic model complexes indeed confirmed the computational prediction and found sluggish reactivity by Cpd 0 as compared to Cpd I [32].

Since then research has moved to alternative oxidants that could act as a “second oxidant” in P450 catalysis. In this respect, van Eldik and co-workers used biomimetic models of Cpd I and Cpd 0 as well as the one-electron reduced form of Cpd I, designated Cpd II [33,34,35]. They studied different oxidative processes (epoxidation, sulfoxidation, hydroxylation, O–H hydrogen atom abstraction, and hydride-transfer reactions) of Cpd I, Cpd II and Cpd 0 toward a variety of substrates. Interestingly, it was found that Cpd II exhibits competent reactivity in O–H hydrogen atom abstraction reactions, whereas it is the most efficient oxidant in hydride-transfer processes [33]. A recent computational study from our groups reasoned that an additional reduction step of Cpd I to give Cpd II would keep the oxidant active in hydrogen atom abstraction reactions, although probably with lesser oxidative power than Cpd I [36]. Furthermore, due to the rising interest in the reactivity of Cpd II, other computational studies that compare the reactivity of Cpd I and Cpd II mimics in other oxidation processes (aldehyde oxidation and alcohol oxidation) [37] or that exclusively study the reactivity pattern of Cpd II [38] have also been reported. In this work, we expand on the studies reported in Ref [36] and investigate whether Cpd II could be a viable oxidant of O–H hydrogen atom abstraction reactions. In particular, we combine calculations on synthetic model complexes with QM/MM studies on the full enzyme that take the effect of the protein into consideration. Specifically, we compare the reactivity of Cpd I and Cpd II as oxidants.

## 2. Results

**Figure 3:**
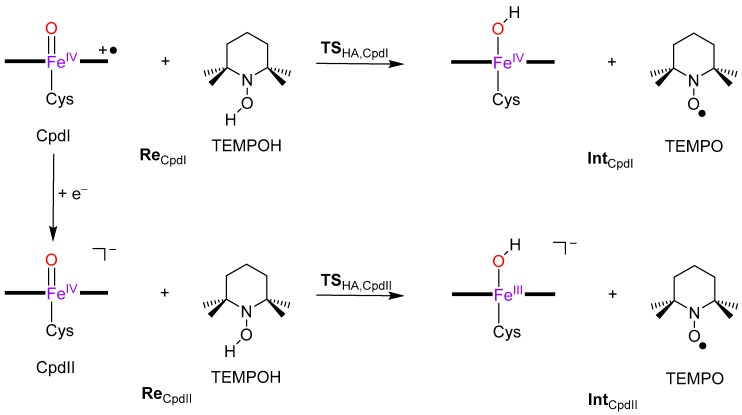
Hydrogen atom abstraction mechanisms with definition of the labels.

To understand the reactivity differences of Cpd I and Cpd II, we decided to do a computational study and used two approaches, namely (1) density functional theory (DFT) studies on enzyme active site models and (2) quantum mechanics/molecular mechanics (QM/MM) calculations on the full protein with solvent layer. Figure 3 displays the calculated reaction mechanism and the definition of the individual structures. As previous work [36] showed that Cpd II may have difficulties with radical rebound steps, we decided to use TEMPOH (2,2,6,6-tetramethyl-piperidine-1-ol) as a substrate as it is a one-electron transfer substrate only. The reaction starts with isolated reactants, i.e., Cpd I + TEMPOH (**Re**_CpdI_) or Cpd II + TEMPOH (**Re**_CpdII_), and proceeds via hydrogen atom abstraction via transition state **TS**_HA_ to form a radical intermediate **Int**, which is an iron(IV)-hydroxo heme with TEMPO^•^ or iron (III)-hydroxo heme with TEMPO^•^. In a subsequent step two TEMPO^•^ radicals pair up to form a dimer.

We initially studied the reaction described in Figure 3 with a gas-phase DFT model (Section 2.1) but followed it up with a QM/MM study (Section 2.2) that takes the shape and size of the protein into consideration.

### 2.1. DFT on Model Complexes

**Figure 4:**
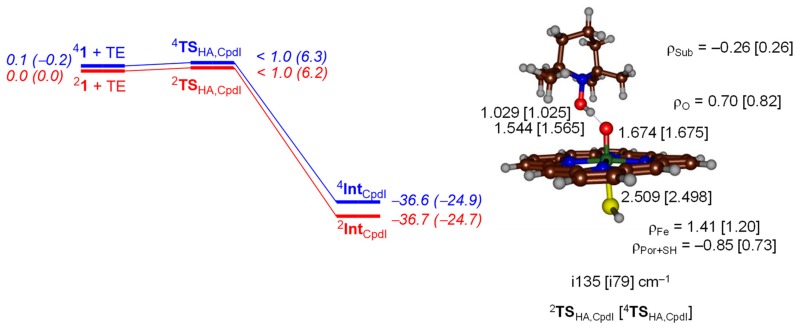
Potential energy landscape with energies in kcal·mol^−1^ calculated at UB3LYP/BS2//UB3LYP/BS1 including solvent and zero-point energy (ZPE) corrections for hydrogen atom abstraction from TEMPOH by ^4,2^Cpd I. Values in parenthesis are solvent corrected free energies. Optimized geometries of the transition states are given on the right-hand-side with bond lengths in angstroms, group spin densities (ρ) in atomic units, and the imaginary frequency in wavenumbers.

We started with a small gas-phase DFT model that contains an iron (IV)-oxo porphyrin cation radical model used previously [39,40,41] that has all substituents on the porphyrin replaced by hydrogen atoms and an axial thiolate ligand. We calculated the mechanism on the lowest lying doublet and quartet spin state surfaces. Figure 4 displays the potential energy profile for hydrogen atom abstraction by ^4,2^Cpd I (^4,2^**1**) or ^4,2^[Fe^IV^(O)(Por^+•^)SH] from TEMPOH. As can be seen on both spin states, the hydrogen atom abstraction barrier is negligible and collapses to the iron (IV)-hydroxo product rapidly. The structure shows short TEMPO–H and long H–OFe distances and as such the transition states are early on the potential energy landscape. These distances match with previously reported hydrogen atom abstraction structures by P450 Cpd I well [42], although different substrates were investigated.

Subsequently, we calculated the same mechanism but from Cpd II (^5,3^**2**) on the triplet and quintet spin states. On the triplet spin state, a small barrier is encountered, whereas it is barrierless on the somewhat higher lying quintet spin state. Therefore, both Cpd I and Cpd II should be able to easily activate TEMPOH at room temperature. The driving force is slightly less exothermic for Cpd II than for Cpd I and follows the order of the hydrogen atom abstraction barriers well. The geometries of the Cpd I and Cpd II hydrogen atom abstraction transition states are dramatically different. As mentioned above for Cpd I the transition states are early with short TEMPO–H distances; however, for the Cpd II transition states these are well larger, in particular for ^3^**TS**_HA,CpdII_ where we find a TEMPO–H distance of 1.329 Å as compared to a value of 1.029 Å for ^2^**TS**_HA,CpdI_. At the same time the accepting O–H distance is considerably shorter for the Cpd II transition states as compared to the ones for Cpd I.

**Figure 5:**
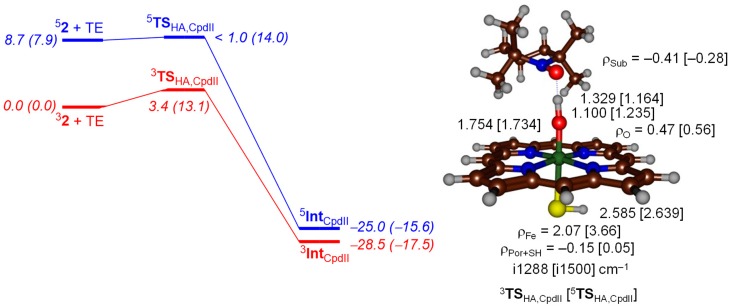
Potential energy landscape with energies in kcal·mol^−1^ calculated at UB3LYP/BS2//UB3LYP/BS1 including solvent and ZPE corrections for hydrogen atom abstraction from TEMPOH by ^3,5^Cpd II. Values in parenthesis are solvent corrected free energies. Optimized geometries of the transition states are given on the right-hand-side with bond lengths in angstroms, group spin densities (ρ) in atomic units and the imaginary frequency in wavenumbers.

**Figure 6:**
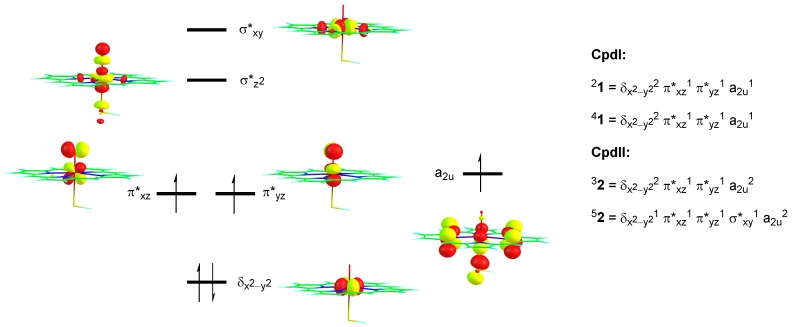
Orbital diagram of Cpd I and Cpd II with occupation in the relevant spin states.

The most dramatic difference, as seen from Figure 4 and Figure 5 is that the substrate approach on the oxo group is from the side for Cpd I, while it is from the top in Cpd II. The substrate approach previously [43,44,45,46,47] was correlated with the electron transfer process that happens in the transition state. In Cpd I the oxidant has electronic configuration of δ_x2-y2_^2^ π*_xz_^1^ π*_yz_^1^ a_2u_^1^, whereby the three unpaired electrons are either ferromagnetically coupled into a quartet spin state or have the heme a_2u_ electron with down-spin and the π*_xz_ and π*_yz_ electrons with up-spin into an overall doublet spin state. Figure 6 gives the orbital shapes and Cpd I and Cpd II occupation numbers. The lowest energy orbital of the set shown in Figure 6 is the δ_x2-y2_ orbital, which is non-bonding and in the plane of the porphyrin ring. The degenerate π*_xz_ and π*_yz_ molecular orbitals are higher in energy for the antibonding interactions of metal and oxo group through 3d and 2p atomic orbital components in these planes. Two virtual orbitals complete the set of metal-type orbitals, namely the σ*_z2_ and the σ*_xy_. The former is the σ*_z2_ antibonding orbital for the metal with oxo interaction, whereas the σ*_xy_ orbital represents the antibonding interactions of the metal with the heme nitrogen atoms. The heme also has several high-lying π*-type orbitals and one of them, the a_2u_ orbital, is close in energy to the π*_xz_ and π*_yz_ orbitals and is singly occupied. Upon reduction of Cpd I to Cpd II, the additional electron fills the a_2u_ orbital with a second electron in the triplet spin state, whereas in the quintet spin state also a promotion from δ_x2-y2_ to σ*_xy_ takes place. Consequently, the electronic configuration of ^3^Cpd II (^3^**2**) is δ_x2-y2_^2^ π*_xz_^1^ π*_yz_^1^ a_2u_^2^ and ^5^Cpd II (^5^**2**) is δ_x2-y2_^1^ π*_xz_^1^ π*_yz_^1^ σ*_xy_^1^ a_2u_^2^.

Hydrogen atom abstraction from substrate by Cpd I, typically fills the a_2u_ orbital with a second electron. This usually [48,49,50,51,52] creates a hydrogen atom abstraction structure with a Fe–O–H angle of around 120°, i.e., gives a side-on approach of substrate on oxidant. Indeed, the transition states shown in Figure 4 for the reactivity of Cpd I show this side-on approach in line with the literature. An iron (IV)-hydroxo (porphyrin) complex is then formed in the doublet and quartet spin states with orbital occupation δ_x2-y2_^2^ π*_xz_^1^ π*_yz_^1^ a_2u_^2^ Φ_Sub_^1^, whereby Φ_Sub_ is the radical on the substrate which is up-spin in the quartet spin state but down-spin in the doublet spin state. Indeed, the group spin densities show that the spin on the porphyrin ring drops from −1/ + 1 in ^2^Cpd I/^4^Cpd I in the reactants to −0.85/0.73 in ^2^**TS**_HA,CpdI_/^4^**TS**_HA,CpdI_, respectively (Figure 4). These values drop further to −0.15 and −0.13 for the radical intermediates with concomitant increase of spin density on the substrate moiety.

The reaction of Cpd II with TEMPOH also leads to hydrogen atom abstraction and the donation of another electron into the oxidant set of orbitals. The lowest available virtual orbital for Cpd II is the σ*_z2_ orbital, which is located along the Fe–O bond. This will lead to an iron (III)-hydroxo(porphyrin) with orbital occupation of δ_x2-y2_^2^ π*_xz_^1^ π*_yz_^1^ σ*_z2_^1^ a_2u_^2^ Φ_Sub_^1^ in the triplet spin state. The substrate radical in the triplet spin state is down-spin, while the metal-based orbitals have an unpaired up-spin electron. The transition state for hydrogen atom abstraction (Figure 5) indeed gives negative spin density on the substrate group (−0.41) and a large spin density on the FeO group (2.54) and implicates electron transfer of an up-spin electron from the substrate into the σ*_z2_ orbital along the iron-oxo bond. In the quintet spin state, the σ*_xy_ orbital is also filled with one electron and the hydrogen atom abstraction creates a quintet spin iron (III)-hydroxo (porphyrin) complex with orbital occupation δ_x2-y2_^1^ π*_xz_^1^ π*_yz_^1^ σ*_z2_^1^ σ*_xy_^1^ a_2u_^2^ Φ_Sub_^1^. The group spin densities show negative spin density arising on the substrate moiety (−0.28 in ^5^**TS**_HA,CpdII_), while the spin on the FeO group increases to 4.22 (Figure 5). Thus, in both triplet and quintet Cpd II, the electron transfer from substrate to oxidant is into the σ*_z2_ orbital, which is located along the Fe–O axis. Therefore, the hydrogen atom abstraction is aligned with this axis and we see the substrate approaching from the top. Consequently, the structures of the hydrogen atom transition states depend on the electron transfer processes, as seen previously for iron (IV)-oxo intermediates.

### 2.2. QM/MM Studies

**Figure 7:**
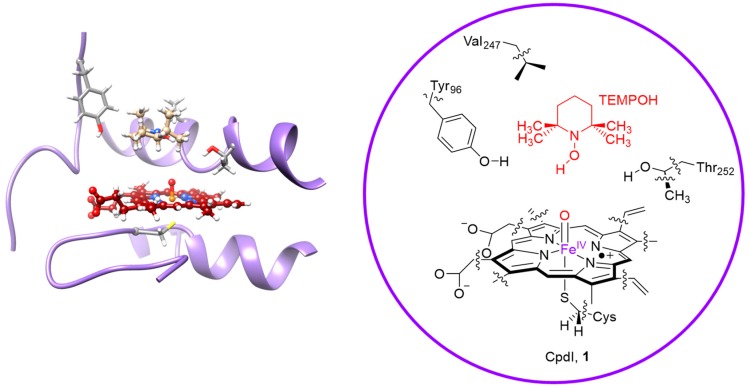
Groups included in the QM region of the QM/MM calculations. Wiggly lines represent bonds between the QM and MM region that were modelled using the link atom approach.

As shown in the previous section, the hydrogen atom abstraction transition states by Cpd I and Cpd II models are very different. While in the former case the substrate approaches from the side, in the latter case a top-approach is found. Obviously in enzymatic systems not always an ideal approach is possible due to the size and shape of the substrate binding pocket and often it is seen that enzymes catalyze stereoselective and regiospecific reaction mechanisms [53,54,55,56]. Furthermore, sometimes small model complexes do not reproduce the correct charge and spin distributions of the real P450 active site due to the absence of, for example, hydrogen bonding interactions to the axial thiolate group [57,58,59,60,61], and consequently full enzymatic approaches are more realistic. Indeed, it was shown previously that hydrogen bonding interactions toward thiolate can affect its charge and pull radical density away from the heme. Therefore, we decided to explore the hydrogen atom abstraction pathway by P450 Cpd I and Cpd II using QM/MM methods. Details of the methodology and set-up have been reviewed and explained in detail elsewhere [62,63]. In general, we take a pdb file from the literature, here 4JWU [64] was used, and modify it to create reactant complexes of either Cpd I + TEMPOH or Cpd II + TEMPOH. From the molecular dynamics simulation several snapshots were tested as starting structures for the QM/MM calculations. The choice of QM region is carefully considered and based on the bonds that are broken, hydrogen bonding interactions and salt bridges. A detailed explanation on how to set up a QM/MM calculation and what to consider as QM region is given in Ref [62]. The QM region used in the QM/MM calculations is displayed in Figure 7 and contained the heme without side chains, methylthiolate for the cysteinate axial ligand and the full TEMPOH substrate. In addition, the aromatic side-chain of Tyr_96_, the side chain of Val_247_ and a methanol group representing the Thr_252_ side chain were included in the QM region. In all places where the border between the QM and MM regions contains a chemical bond, we inserted link atoms (hydrogens).

**Figure 8:**
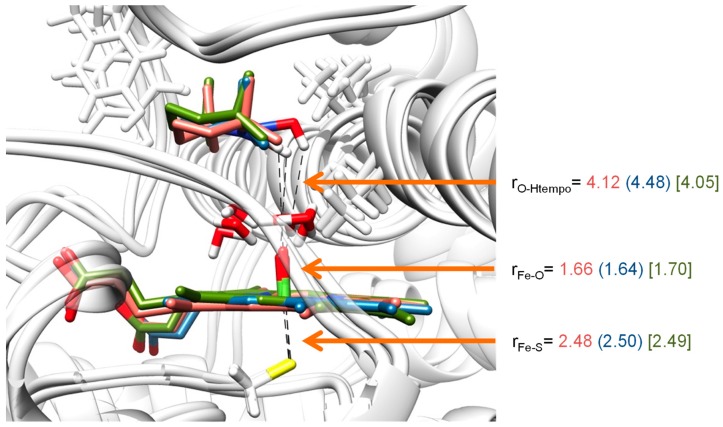
QM/MM optimized geometries of the reactant complexes ^2^**1** as obtained with UB3LYP/BS3:Charmm. Bond lengths are in angstroms. Data presented are for snapshots Sn_250_ (salmon), Sn_400_ (blue) and Sn_500_ (green).

Figure 8 displays the optimized geometries of ^2^Cpd I as calculated with QM/MM for snapshots Sn_250_, Sn_400_ and Sn_500_ (see Material and Methods section for detailed information). The Fe–O distances vary slightly and range from 1.64 Å for Sn_400_ to 1.70 Å for Sn_500_, while the Fe–S distances are within 0.02 Å of each other. These distances are in agreement with the results of the small model complexes where generally Fe–O distances of ca. 1.64–1.66 Å and Fe–S distances of 2.50 Å were obtained for small model complexes as well as previous QM/MM studies on P450 Cpd I structures [65,66,67]. Furthermore, the experimental crystal structure coordinates and spectroscopic analysis also found Fe–O distances of Cpd I and Cpd II of heme enzymes in close proximity of our computational distances for related heme proteins [68].

**Figure 9:**
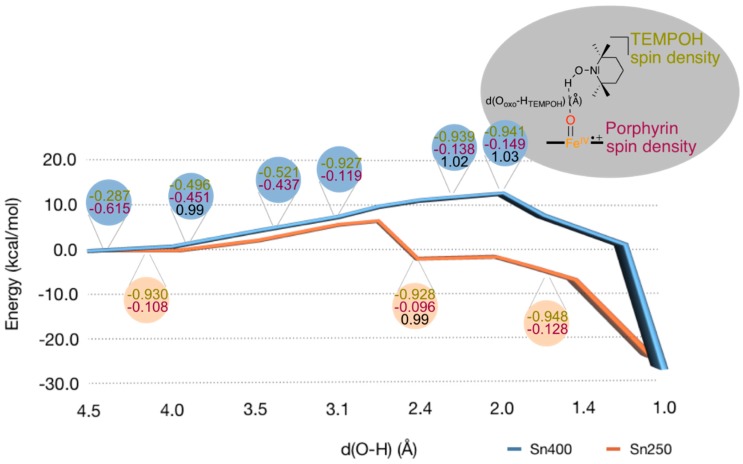
QM/MM calculated geometry scans for substrate approach on the iron (IV)-oxo species of ^2^Cpd I as calculated for snapshots Sn_250_ (salmon) and Sn_400_ (blue). Each point represents a full geometry optimization with fixed FeO···HO distance between oxo and TEMPOH proton. In the rounded boxes, Mulliken spin densities (in atomic units) of the TEMPOH substrate (green) and the porphyrin ring (plum) are represented, as well as the O_oxo_-H_TEMPOH_ distance (Å) for selected structures (black).

Subsequently, we explored the hydrogen atom abstraction barrier from TEMPOH by Cpd I using QM/MM for snapshots Sn_250_ and Sn_400_, see Figure 9. Initially, we performed a geometry scan for the approach of TEMPOH to Cpd I by freezing the FeO–H distance at each step and reoptimizing the rest of the coordinates. The results in Figure 9 show that cleaving the O–H bond of TEMPOH by Cpd I behaves in a different way than that found by DFT on model compounds. In particular, the cleavage has a barrier of about 10 kcal mol^−1^ that builds up during TEMPOH’s approach to Cpd I and which is due to the rearrangement of the protein environment. During the approach, between the FeO·H_TEMPOH_ distance of 2.00 to 4.00 Å, an electron transfer from TEMPOH to the porphyrin group takes place that creates the radical TEMPOH^+•^ cation radical and an [Fe^IV^ = O (Por) (Cys)]^−^ compound, i.e., Cpd II. Thus, a long-range electron transfer by TEMPOH to Cpd I gives Cpd II and oxidized substrate (TEMPOH^+•^). The electron transfer, which is expected to have a very low barrier, eases the approach of TEMPOH and reduces a hypothetical higher barrier. At the FeO–H_TEMPOH_ distance of 2.00 Å, the TEMPOH’s O–H distance is still 1.03 Å and, from this distance, the scan energy starts decreasing until products are reached. Thus, TEMPOH’s O–H bond cleavage takes place in a second barrierless stage where a proton is abstracted from TEMPOH^+•^, creating the TEMPO^•^ radical. Consequently, the global process that occurs in the protein is better described as a proton-coupled-electron transfer (PCET), unlike the hydrogen atom transfer (HAT) process that takes place in model complexes.

From the maximum points in the geometry scans of Figure 9, we attempted to locate transition states. However, the energetic fluctuation during the scan does not represent a barrier of a chemical reaction through bond breaking and/or bond forming, but is related to the structural reorganization in the substrate binding-pocket. In particular, during approach of the substrate on the oxidant, hydrogen bonding interactions are weakened with one group and strengthened with other groups. In addition, water molecules migrate in the substrate binding-pocket and also undergo hydrogen bonding interactions. As such caution should be taken when interpreting these geometry scans. Specifically, the maximum energy points are at a relatively long O–H distance of around 2 Å for Sn_400_ and 2.8 Å for Sn_250_. The O–H distances in the scan maxima structures are well longer than normally found in hydrogen atom abstraction transition states that are in the order of 1.2–1.4 Å [42,47,48,49,69,70,71,72,73]. Therefore, no proper hydrogen atom abstraction transition states could be characterized. Since the scan energy profile at short distances gives little evidence of a proper hydrogen atom abstraction barrier, it implies that also in the protein the hydrogen atom abstraction barrier from TEMPOH by Cpd I is negligible. Consequently, the small model complex captures the potential energy profile of hydrogen atom abstraction quite well and predicts negligible hydrogen atom abstraction barriers probably because the substrate fits into the substrate binding pocket well and can approach the oxidant under the ideal angle.

In summary, the hydrogen atom abstraction by Cpd I of TEMPOH encounters a barrier due to electron transfer and protein movement and, of course, varies with the snapshot chosen. During this process the O–H distance in TEMPOH remains short (see black data in the rounded boxes in Figure 9). The actual hydrogen atom abstraction to oxidant, however, is virtually barrierless and no transition states could be characterized. This process is the same regardless of the snapshot we investigated.

**Figure 10:**
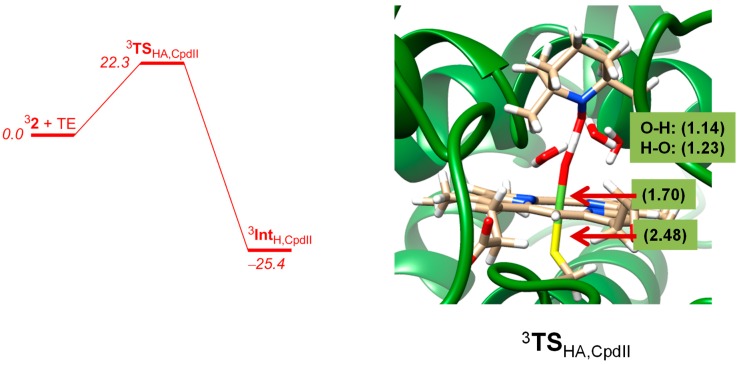
QM/MM calculated potential energy profile of hydrogen atom abstraction from TEMPOH by ^3^Cpd II as obtained with UB3LYP/BS3:Charmm. The optimized geometry of the transition state for Sn_400_ is shown with bond lengths in angstroms.

Next, we investigated the hydrogen atom abstraction from TEMPOH by ^3^Cpd II and the results are given in Figure 10. For Cpd II a proper hydrogen atom abstraction transition state could be located with an energy barrier of 22.3 kcal mol^−1^ with respect to a reactant complex. Again, the QM/MM barrier is higher in energy than the gas-phase barrier due to the protein environment that prevents an ideal approach of substrate to oxidant and thereby raises the barriers with respect to the gas-phase model complexes. Consequently, a tight binding pocket will have a barrier-raising effect but on the other hand may trigger a regioselective or stereospecific reaction path. The enzyme, therefore, balances a regioselective reaction mechanism at a cost of a slower reaction rate. For Cpd II the approach of substrate on the oxidant also gives a geometry scan that fluctuates due to the forming and breaking of hydrogen bonding interactions. However, upon close distance a high barrier appears for the bond breaking and forming processes during the reaction.

The QM/MM optimized geometry for hydrogen atom abstraction from TEMPOH by Cpd II is shown in Figure 10. The structure is reactant-like with short TEMPO–H distance of 1.14 Å, whereas in the DFT model a value of 1.33 Å was found. Similarly, the FeO–H distance is relatively long in QM/MM (1.23 Å) and much shorter in the gas-phase model (1.10 Å). These differences in optimized geometry are due to several hydrogen bonding interactions surrounding the iron(IV)-oxo and substrate groups and, in particular, three crystal water molecules are highlighted in Figure 10 that are involved in hydrogen bonding interactions to the oxo as well as amide groups and thereby affect the hydrogen transfer mechanism. Obviously, hydrogen bonding interactions from crystal water molecules in the substrate binding pocket have strong effects on the position of the substrate with respect to the oxidant as well as a charge-donation that influences transition state structures and ultimately barrier heights. In previous studies the effect of hydrogen bonding interactions to the oxo group were shown to raise hydrogen atom abstraction barriers significantly [74] in line with the difference between DFT and QM/MM as seen here.

## 3. Discussion

In this work a series of DFT and QM/MM results are presented on the reactivity of cytochrome P450 Cpd I and Cpd II intermediates with a model substrate, namely TEMPOH. It is seen that the lowest reaction barriers are obtained with Cpd I, although those found for Cpd II are also accessible at room temperature depending on the method and model. As such both Cpd I and Cpd II should be able to activate substrates with weak or medium strength C–H bonds efficiently. In the following, we will try to rationalize the reactivity differences and particularly focus on the thermodynamics of the reactions.

In principle, the hydrogen atom abstraction reaction from a substrate (TEMPOH) by Cpd I or [Fe^IV^(O)(heme^+•^)Cys] gives an iron(IV)-hydroxo species and a radical rest group (TEMPO^•^) as described with Equation (1). Thermodynamically, the energy for Equation (1) (∆H_Eq1_) is the difference in energy of the bond dissociation energy of the substrate O–H bond that is broken (BDE_OH, TE_), Equation (2), and the bond dissociation energy of the O–H bond of the iron (IV)-hydroxo complex that is formed (BDE_OH,CpdI_), Equation (3) [42,48,49,75,76,77,78,79,80,81,82]. As such the reaction energy for hydrogen atom abstraction from substrate by Cpd I (Equation (4)) can be described by individual bond dissociation energies BDE_OH,TE_ and BDE_OH,CpdI_.

[Fe^IV^(O)(heme^+•^)Cys] + TEMPOH → [Fe^IV^(OH)(heme)Cys] + TEMPO^•^ (1)

TEMPOH → TEMPO^•^ + H^•^ + BDE_OH,TE_ (2)

[Fe^IV^(OH)(heme)Cys] → [Fe^IV^(O)(heme ^+•^)Cys] + H^•^ + BDE_OH,CpdI_ (3)

∆H_Eq1_ = BDE_OH,TE_ − BDE_OH,CpdI_ (4)

To this end we calculated the BDE_OH_ values of TEMPOH, the iron (IV)-hydroxo (**2H^+^**) and iron (III)-hydroxo complexes, i.e., BDE_OH,TE_, BDE_OH,CpdI_ and BDE_OH,CpdII_, respectively. TEMPOH has a very weak O–H bond strength of 56.4 kcal mol^−1^. Typical values for aliphatic C–H bond strengths calculated at the same level of theory are 81.9 kcal mol^−1^ for abstraction of a hydrogen atom from the α-position of ethylbenzene and 93.3 kcal mol^−1^ for cyclohexane [36]. Clearly, the O–H bond of TEMPOH is weak and it should not cost oxidants much energy to abstract its hydrogen atom. Indeed, a negligible barrier is found for Cpd I and a small barrier for Cpd II is found in agreement with the strength of the O–H bond of the substrate. Based on the BDE_OH_ values [36], we predict hydrogen atom abstraction driving forces for Cpd I of –35.3 kcal mol^−1^ and for Cpd II of −31.7 kcal mol^−1^. These values are in good quantitative agreement with the energy differences between reactants and intermediates seen in Figure 4 and Figure 5 above. The small energetic differences result from dispersion and intermolecular interaction energies.

**Figure 11:**
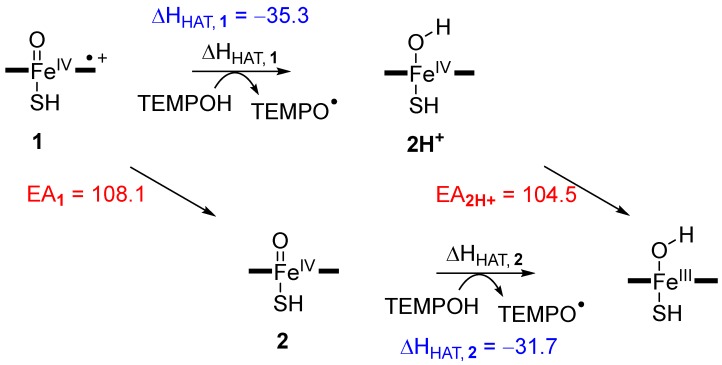
Thermochemical reaction steps for hydrogen atom abstraction and electron transfer from Cpd I and Cpd II. Values are in kcal mol^−1^ calculated with a DFT model complex at UB3LYP/BS2 + solvent//UB3LYP/BS1 level of theory.

Figure 11 summarizes the thermochemistry of possible hydrogen atom abstraction and electron transfer processes for both Cpd I and Cpd II. Pathways from left to right represent hydrogen atom abstraction from TEMPOH that give the iron (IV/III)-hydroxo complexes. Also given on the diagonal axis in Figure 11 are the electron affinities (EA) of Cpd I and protonated Cpd II (**2H^+^**). Note that the electron affinity of Cpd II is 50.1 kcal mol^−1^ using the same computational methods.

Thus, the BDE_OH_ of Cpd I is larger than that of Cpd II and therefore, Cpd I will react with lower hydrogen atom abstraction barriers with substrates. However, the energy difference is not dramatic, i.e., just a few kcal mol^−1^ in energy, so that both oxidants should be able to activate substrates as easily. Consequently, if excess reduction appears in P450 isozymes and Cpd II is formed, the enzyme will still be active in substrate activation processes.

## 4. Materials and Methods

Two different approaches were used in this work, namely (1) DFT studies on enzyme active site models and (2) QM/MM calculations on a full enzymatic structure of a P450 isozyme. These QM/MM methods have been described in details elsewhere [55,56,62,63,83,84,85,86,87,88,89,90,91] and were extensively benchmarked and calibrated against experimental data. In particular, rate constants of small model complexes were reproduced excellently as compared to experimental data for oxygen atom transfer by iron (IV)-oxo and iron (IV)-imido species [92,93,94,95,96,97,98,99,100,101]. In addition, reduction potentials were reproduced well [102,103]. Furthermore, previous QM/MM studies predicted the correct regio- and chemoselectivities of substrate activation in highly selective enzyme reactions [55,63].

### 4.1. DFT Model Complexes

DFT model complexes were calculated in the Gaussian-09 program package [104] and investigated with density functional theory methods. Geometry optimizations, frequency calculations, geometry scans and intrinsic reaction coordinate profiles were performed at the unrestricted B3LYP level of theory [105,106] and utilized an LACVP basis set with core potential [107] on iron and 6-31G on the rest of the atoms; basis set BS1. Energies were improved at the single point level of theory with an LACV3P+ basis set on iron and 6-311+G* on the rest of the atoms; basis set BS2. Previously, we showed that UB3LYP/BS2 optimized geometries and potential energy landscapes were within a few tenths of a kcal mol^−1^ from those obtained at UB3LYP/BS2//UB3LYP/BS1 [42,78,100] Moreover, spin state orderings were reproduced well with this method [108]. In addition, the calculations included the polarized continuum model with a dielectric constant of ε = 5.697, which is a typical value for the active site within proteins. Note that earlier test calculations with a range of dielectric constants only gave minor changes to spin state orderings and relative energies and the major difference found was upon changing from the gas-phase to a solvent model [109].

The chemical model was based on a typical P450 active site structure and included an iron embedded in protoporphyrin IX (Por) without substituents on the periphery. The axial cysteinate ligand of iron was abbreviated to thiolate as that was shown to be a better mimic than methylthiolate [110,111].

### 4.2. QM/MM Set Up

**Figure 12:**
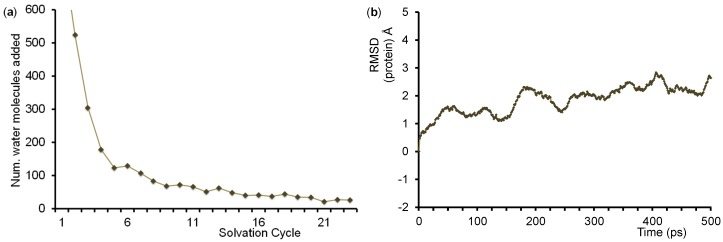
QM/MM set-up related graphics. (**a**) Number of water molecules added in each cycle of the hydration procedure to solvate the system. The number of water molecules added in step 1 using a 40 Å sphere box was 6454; (**b**) Changes in the root-mean-square deviation (RMSD), in Å, of the protein atomic positions over the course of the molecular dynamics simulation (picoseconds).

The QM/MM model was built from the 4JWU protein databank (pdb) file [64], which is a bacterial (*Pseudomonas putida*) P450 isozyme crystal structure that contains the homodimer of the Cytochrome P450 domain with its redox partner putidaredoxin [112]. We removed the redox partner and selected a complete single strand (both are identical). We inserted TEMPOH substrate manually into the enzyme structure on the distal site of the heme, and, in addition, replaced the iron(III)-water group by an iron(IV)-oxo species with an Fe-O starting bond distance of 1.65 Å as is typically found in heme Cpd I structures in calculations [113,114]. Subsequently, hydrogen atoms were added to the structure using the online tool pdb2pqr at pH = 7 [115]. All acidic and basic protein groups were visually inspected for having the correct protonation state and in our QM/MM models all carboxylate groups (Asp/Glu) were found to be deprotonated and all Arg and Lys amino acid side chains were protonated. Note that this particular P450 isozyme contains no histidine residues in the protein. In the final stage of the structure preparation, we performed an iterative solvation procedure (left graphic in Figure 12) and added TIP3P water molecules to obtain a final structure containing 32,447 atoms including 8657 water molecules. The structure was neutralized with ten Mg^2+^ and four Cl^−^ ions on the surface of the protein. In the next stages of the QM/MM set up we applied a stepwise heating protocol to 300 K using the Charmm force field [116] followed by full equilibration whereby the protein backbone was kept fixed. Thereafter, a full molecular dynamics (MD) simulation was run for 500 ps, see Figure 12 (right), from which we selected three low-energy snapshots after 250, 400 and 500 ps for the actual QM/MM calculations: Sn_250_, Sn_400_ and Sn_500_.

The model was split into a QM and MM region, whereby the QM part was described by density functional theory, whereas the MM part was calculated with the Charmm forcefield [116]. The QM region was calculated at the UB3LYP/BS3 level of theory [105,106] with BS3 representing a def-SVP basis set on all atoms [117]. The QM and MM regions are interfaced by ChemShell [118] through DL-Poly [119], whereby the charges of the MM region were incorporated into the QM Hamiltonian through electrostatic embedding [120]. The boundary of the QM and MM region was described through the Link-atom approach [121]. Geometries were optimized in QM/MM without constraints, while the MM region had a flexible component of 5 Å around the heme and substrate moieties, while all other atoms in the MM region were fixed in the original snapshot orientation. Cpd I and Cpd II were calculated in the doublet and triplet spin states, respectively.

The geometry scan for the hydrogen atom transfer from TEMPOH to the oxidant was performed through stepwise geometry optimizations at QM/MM level of theory with fixed O–H bond distances, while all other degrees of freedom were minimized. The maxima of these scans were subjected to a full transition state search.

## 5. Conclusions

In this work a computational study is presented in the reaction of P450 Cpd I and Cpd II with TEMPOH using a combined density functional theory and QM/MM approach. These studies investigate O–H hydrogen atom abstractions of the two potential oxidants and discuss their feasibility in an enzymatic setting. The results show that both Cpd I and Cpd II can react with substrates with weak O–H bonds. Interestingly, Cpd I in the protein reacts via a proton-coupled electron transfer process rather than hydrogen atom abstraction. Thus, the protein enables a long-range electron transfer from substrate to Cpd I followed by a proton transfer. These studies show distinct differences between gas-phase DFT models and protein structures. Overall, both Cpd I and Cpd II are found to be suitable oxidants for TEMPOH activation.

## References

[1] Sono M., Roach M.P., Coulter E.D., Dawson J.H. (1996). Heme-containing oxygenases. Chem. Rev..

[2] Ortiz de Montellano P.R. (2004). Cytochrome P450: Structure, Mechanism, and Biochemistry.

[3] Meunier B., de Visser S.P., Shaik S. (2004). Mechanism of oxidation reactions catalyzed by cytochrome P450 enzymes. Chem. Rev..

[4] Denisov I.G., Makris T.M., Sligar S.G., Schlichting I. (2005). Structure and chemistry of cytochrome P450. Chem. Rev..

[5] Munro A.W., Girvan H.M., McLean K.J. (2007). Variations on a (t)heme—novel mechanisms, redox partners and catalytic functions in the cytochrome P450 superfamily. Nat. Prod. Rep..

[6] Ortiz de Montellano P.R. (2010). Hydrocarbon hydroxylation by cytochrome P450 enzymes. Chem. Rev..

[7] Cai H., Guengerich F.P. (2001). Reaction of trichloroethylene and trichloroethylene oxide with cytochrome P450 enzymes: Inactivation and sites of modification. Chem. Res. Toxicol..

[8] Grogan G. (2011). Cytochromes P450: Exploiting diversity and enabling application as biocatalysts. Curr. Opin. Chem. Biol..

[9] De Visser S.P., Kumar D. (2011). Iron-Containing Enzymes: Versatile Catalysts of Hydroxylation Reactions in Nature.

[10] Groves J.T. (2003). The bioinorganic chemistry of iron in oxygenases and supramolecular assemblies. Proc. Natl. Acad. Sci. USA.

[11] Watanabe Y., Nakajima H., Ueno T. (2007). Reactivities of oxo and peroxo intermediates studied by hemoprotein mutants. Acc. Chem. Res..

[12] De Visser S.P., Nam W., Kadish K.M., Smith K.M., Guilard R. (2010). High-valent iron-oxo porphyrins in oxygenation reactions. Handbook of Porphyrin Science.

[13] Huang X., Groves J.T. (2018). Oxygen activation and radical transformations in heme proteins and metalloporphyrins. Chem. Rev..

[14] Davydov R., Makris T.M., Kofman V., Werst D.E., Sligar S.G., Hoffman B.M. (2001). Hydroxylation of camphor by reduced oxy-cytochrome P450cam: Mechanistic implications of EPR and ENDOR studies of catalytic intermediates in native and mutant enzymes. J. Am. Chem. Soc..

[15] Gelb M.H., Heimbrook D.C., Malkonen P., Sligar S.G. (1982). Stereochemistry and deuterium isotope effects in camphor hydroxylation by the cytochrome P450_cam_ monoxygenase system. Biochemistry.

[16] Makris T.M., von Koenig K., Schlichting I., Sligar S.G. (2007). Alteration of P450 distal pocket solvent leads to impaired proton delivery and changes in heme geometry. Biochemistry.

[17] Poulos T.L., Finzel B.C., Howard A.J. (1987). High-resolution crystal structure of cytochrome P450cam. J. Mol. Biol..

[18] Cong Z., Shoji O., Kasai C., Kawakami N., Sugimoto H., Shiro Y., Watanabe Y. (2015). Activation of Wild-Type Cytochrome P450BM3 by the Next Generation of Decoy Molecules: Enhanced Hydroxylation of Gaseous Alkanes and Crystallographic Evidence. ACS Catal..

[19] Butler C.R., Ogilvie K., Martinez-Alsina L., Barreiro G., Beck E.M., Nolan C.E., Atchison K., Benvenuti E., Buzon L., Doran S. (2017). Aminomethyl-derived beta secretase (BACE1) inhibitors: Engaging Gly230 without an anilide functionality. J. Med. Chem..

[20] Kumar D., Hirao H., de Visser S.P., Zheng J., Wang D., Thiel W., Shaik S. (2005). New features in the catalytic cycle of cytochrome P450 during the formation of Compound I from Compound 0. J. Phys. Chem. B.

[21] Shaik S., Kumar D., de Visser S.P., Altun A., Thiel W. (2005). Theoretical perspective on the structure and mechanism of cytochrome P450 enzymes. Chem. Rev..

[22] Balding P.R., Porro C.S., McLean K.J., Sutcliffe M.J., Maréchal J.-D., Munro A.W., de Visser S.P. (2008). How do azoles inhibit cytochrome P450 enzymes? A density functional study. J. Phys. Chem. A.

[23] Davydov R., Perera R., Jin S., Yang T.-C., Bryson T.A., Sono M., Dawson J.H., Hoffman B.M. (2005). Substrate modulation of the properties and reactivity of the oxy-ferrous and hydroperoxo-ferric intermediates of cytochrome P450_cam_ as shown by cryoreduction-EPR/ENDOR spectroscopy. J. Am. Chem. Soc..

[24] Rittle J., Green M.T. (2010). Cytochrome P450 Compound I: Capture, characterization, and C-H bond activation kinetics. Science.

[25] Ogliaro F., Harris N., Cohen S., Filatov M., de Visser S.P., Shaik S. (2000). A model “rebound” mechanism of hydroxylation by cytochrome P450: Stepwise and effectively concerted pathways, and their reactivity patterns. J. Am. Chem. Soc..

[26] Kamachi T., Yoshizawa K. (2003). A Theoretical study on the mechanism of camphor hydroxylation by Compound I of cytochrome P450. J. Am. Chem. Soc..

[27] Li D., Wang Y., Han K. (2012). Recent density functional theory model calculations of drug metabolism by cytochrome P450. Coord. Chem. Rev..

[28] Vaz A.D.N., Pernecky S.J., Raner G.M., Coon M.J. (1996). Peroxo-iron and oxenoid-iron species as alternative oxygenating agents in cytochrome P450-catalyzed reactions: Switching by Threonine-302 to Alanine mutagenesis of cytochrome P450 2B4. Proc. Natl. Acad. Sci. USA.

[29] Ogliaro F., de Visser S.P., Cohen S., Sharma P.K., Shaik S. (2002). Searching for the second oxidant in the catalytic cycle of cytochrome P450: A theoretical investigation of the iron(III)-hydroperoxo species and its epoxidation pathways. J. Am. Chem. Soc..

[30] Sharma P.K., de Visser S.P., Shaik S. (2003). Can a single oxidant with two spin states masquerade as two different oxidants? A study of the sulfoxidation mechanism by cytochrome P450. J. Am. Chem. Soc..

[31] Porro C.S., Sutcliffe M.J., de Visser S.P. (2009). Quantum mechanics/molecular mechanics studies on the sulfoxidation of dimethyl sulfide by Compound I and Compound 0 of Cytochrome P450: Which is the better oxidant?. J. Phys. Chem. A.

[32] Song W.J., Ryu Y.O., Song R., Nam W. (2005). Oxoiron(IV) porphyrin pi-cation radical complexes with a chameleon behavior in cytochrome P450 model reactions. J. Biol. Inorg. Chem..

[33] Fertinger C., Hessenauer-Ilicheva N., Franke A., van Eldik R. (2009). Direct comparison of the reactivity of model complexes for Compounds 0, I, and II in oxygenation, hydrogen-abstraction, and hydride-transfer processes. Chem. Eur. J..

[34] Fertinger C., Franke A., van Eldik R. (2012). Mechanistic insight from thermal activation parameters for oxygenation reactions of different substrates with biomimetic iron porphyrin models for compounds I and II. J. Biol. Inorg. Chem..

[35] Ji L., Franke A., Brindell M., Oszajca M., Zahl A., van Eldik R. (2014). Combined experimental and theoretical study on the reactivity of Compounds I and II in horseradish peroxidase biomimetics. Chem. Eur. J..

[36] Li X.-X., Postils V., Sun W., Faponle A.S., Solà M., Wang Y., Nam W., de Visser S.P. (2017). Reactivity patterns of (protonated) Compound II and Compound I of Cytochrome P450: Which is the better oxidant?. Chem. Eur. J..

[37] Oszajca M., Franke A., Drzewiecka-Matuszek A., Brindell M., Stochel G., van Eldik R. (2014). Temperature and pressure effects on C–H abstraction reactions involving compound I and II mimics in aqueous solution. Inorg. Chem..

[38] Mallick D., Shaik S. (2017). Kinetic isotope effect probes the reactive spin state, as well as the geometric feature and constitution of the transition state during H-abstraction by heme Compound II complexes. J. Am. Chem. Soc..

[39] De Visser S.P., Tan L.S. (2008). Is the bound substrate in nitric oxide synthase protonated or neutral and what is the active oxidant that performs substrate hydroxylation?. J. Am. Chem. Soc..

[40] İşci Ü., Faponle A.S., Afanasiev P., Albrieux F., Briois V., Ahsen V., Dumoulin F., Sorokin A.B., de Visser S.P. (2015). Site-selective formation of an iron(IV)-oxo species at the more electron-rich iron atom of heteroleptic μ-nitrido diiron phthalocyanines. Chem. Sci..

[41] De Visser S.P., Kumar D., Neumann R., Shaik S. (2004). Computer-generated high-valent iron-oxo and manganese-oxo species with polyoxometalate ligands: How do they compare with the iron-oxo active species of heme enzymes?. Angew. Chem. Int. Ed..

[42] De Visser S.P. (2010). Trends in substrate hydroxylation reactions by heme and nonheme iron(IV)-oxo oxidants give correlations between intrinsic properties of the oxidant with barrier height. J. Am. Chem. Soc..

[43] De Visser S.P. (2006). Propene activation by the oxo-iron active species of taurine/α-ketoglutarate dioxygenase (TauD) enzyme. How does the catalysis compare to heme-enzymes?. J. Am. Chem. Soc..

[44] De Visser S.P. (2006). What factors influence the ratio of C–H hydroxylation versus C=C epoxidation by a nonheme cytochrome P450 biomimetic?. J. Am. Chem. Soc..

[45] Kumar D., Latifi R., Kumar S., Rybak-Akimova E.V., Sainna M.A., de Visser S.P. (2013). Rationalization of the barrier height for para-Z-styrene epoxidation by iron(IV)-oxo porphyrins with variable axial ligands. Inorg. Chem..

[46] Tang H., Guan J., Liu H., Huang X. (2013). Analysis of an alternative to the H-atom abstraction mechanism in methane C-H bond activation by nonheme iron(IV)-oxo oxidants. Dalton Trans..

[47] Quesne M.G., Senthilnathan D., Singh D., Kumar D., Maldivi P., Sorokin A.B., de Visser S.P. (2016). Origin of the enhanced reactivity of μ-nitrido-bridged diiron(IV)-oxo porphyrinoid complexes over cytochrome P450 Compound I. ACS Catal..

[48] De Visser S.P., Kumar D., Cohen S., Shacham R., Shaik S. (2004). A predictive pattern of computed barriers for C–H hydroxylation by Compound I of cytochrome P450. J. Am. Chem. Soc..

[49] Shaik S., Kumar D., de Visser S.P. (2008). A valence bond modeling of trends in hydrogen abstraction barriers and transition states of hydroxylation reactions catalyzed by cytochrome P450 enzymes. J. Am. Chem. Soc..

[50] Kumar D., Tahsini L., de Visser S.P., Kang H.Y., Kim S.J., Nam W. (2009). The effect of porphyrin ligands on the regioselective dehydrogenation versus epoxidation of olefins by oxoiron(IV) mimics of cytochrome P450. J. Phys. Chem. A.

[51] Bernasconi L., Baerends E.J. (2008). The EDTA complex of oxidoiron(IV) as realisation of an optimal ligand environment for high activity of FeO^2+^. Eur. J. Inorg. Chem..

[52] Ye S., Geng C.-Y., Shaik S., Neese F. (2013). Electronic structure analysis of multistate reactivity in transition metal catalyzed reactions: The case of C–H bond activation by non-heme iron(IV)–oxo cores. Phys. Chem. Chem. Phys..

[53] De Visser S.P., Latifi R. (2009). Carbon dioxide, a waste product in the catalytic cycle of α-ketoglutarate dependent halogenases prevents the formation of hydroxylated by-products. J. Phys. Chem. B.

[54] Pratter S.M., Konstantinovics C., DiGiuro C.L.M., Leitner E., Kumar D., de Visser S.P., Grogan G., Straganz G.D. (2013). Inversion of enantio-selectivity of a mononuclear non-heme iron(II)-dependent hydroxylase by tuning the interplay of metal-center geometry and protein structure. Angew. Chem. Int. Ed..

[55] Timmins A., Saint-André M., de Visser S.P. (2017). Understanding how prolyl-4-hydroxylase structure steers a ferryl oxidant toward scission of a strong C–H bond. J. Am. Chem. Soc..

[56] Timmins A., de Visser S.P. (2017). How are substrate binding and catalysis affected by mutating Glu127 and Arg161 in prolyl-4-hydroxylase? A QM/MM and MD study. Front. Chem..

[57] De Visser S.P., Ogliaro F., Sharma P.K., Shaik S. (2002). Hydrogen bonding modulates the selectivity of enzymatic oxidation by P450: A chameleon oxidant behavior of Compound I. Angew. Chem. Int. Ed..

[58] Kumar D., de Visser S.P., Sharma P.K., Hirao H., Shaik S. (2005). Sulfoxidation mechanisms catalyzed by cytochrome P450 and horseradish peroxidase models: Spin selection induced by the ligand. Biochemistry.

[59] Sahu S., Widger L.R., Quesne M.G., de Visser S.P., Matsumura H., Moënne-Loccoz P., Siegler M.A., Goldberg D.P. (2013). Secondary coordination sphere influence on the reactivity of nonheme iron(II) complexes: An experimental and DFT approach. J. Am. Chem. Soc..

[60] Morozov A.N., Pardillo A.D., Chatfield D.C. (2015). Chloroperoxidase-catalyzed epoxidation of cis-β-methylstyrene: NH–S hydrogen bonds and proximal helix dipole change the catalytic mechanism and significantly lower the reaction barrier. J. Phys. Chem. B.

[61] Morozov A.N., Chatfield D.C. (2016). How the proximal pocket may influence the enantiospecificities of chloroperoxidase-catalyzed epoxidations of olefins. Int. J. Mol. Sci..

[62] Quesne M.G., Borowski T., de Visser S.P. (2016). Quantum mechanics/molecular mechanics modelling of enzymatic processes: Caveats and breakthroughs. Chem. Eur. J..

[63] Faponle A.S., Seebeck F.P., de Visser S.P. (2017). Sulfoxide synthase versus cysteine dioxygenase reactivity in a nonheme iron enzyme. J. Am. Chem. Soc..

[64] Tripathi S., Li H., Poulos T.L. (2013). Structural basis for effect or control and redox partner recognition in cytochrome P450. Science.

[65] Schöneboom J.C., Lin H., Reuter N., Thiel W., Cohen S., Ogliaro F., Shaik S. (2002). The elusive oxidant species of cytochrome P450 enzymes: Characterization by combined quantum mechanical/molecular mechanical (QM/MM) calculations. J. Am. Chem. Soc..

[66] Bathelt C.M., Zurek J., Mulholland A.J., Harvey J.N. (2005). Electronic structure of compound I in human isoforms of cytochrome P450 from QM/MM modeling. J. Am. Chem. Soc..

[67] Cantú Reinhard F.G., de Visser S.P. (2017). Oxygen atom transfer using an iron(IV)-oxo embedded in a tetracyclic N-heterocyclic carbene system: How does the reactivity compare to Cytochrome P450 Compound I?. Chem. Eur. J..

[68] Schlichting I., Berendzen J., Chu K., Stock A.M., Maves S.A., Benson D.E., Sweet B.M., Ringe D., Petsko G.A., Sligar S.G. (2000). The catalytic pathway of cytochrome P450cam at atomic resolution. Science.

[69] De Visser S.P., Ogliaro F., Sharma P.K., Shaik S. (2002). What factors affect the regioselectivity of oxidation by cytochrome P450? A DFT study of allylic hydroxylation and double bond epoxidation in a model reaction. J. Am. Chem. Soc..

[70] Sharma P.K., de Visser S.P., Ogliaro F., Shaik S. (2003). Is the ruthenium analogue of Compound I of cytochrome P450 an efficient oxidant? A theoretical investigation of the methane hydroxylation reaction. J. Am. Chem. Soc..

[71] De Visser S.P. (2006). Substitution of hydrogen by deuterium changes the regioselectivity of ethylbenzene hydroxylation by an oxo-iron-porphyrin catalyst. Chem. Eur. J..

[72] Latifi R., Bagherzadeh M., de Visser S.P. (2009). Origin of the correlation of the rate constant of substrate hydroxylation by nonheme iron(IV)-oxo complexes with the bond-dissociation energy of the C–H bond of the substrate. Chem. Eur. J..

[73] Ji L., Faponle A.S., Quesne M.G., Sainna M.A., Zhang J., Franke A., Kumar D., van Eldik R., Liu W., de Visser S.P. (2015). Drug metabolism by cytochrome P450 enzymes: What distinguishes the pathways leading to substrate hydroxylation over desaturation?. Chem. Eur. J..

[74] Latifi R., Sainna M.A., Rybak-Akimova E.V., de Visser S.P. (2013). Does hydrogen bonding-donation to manganese(IV)-oxo and iron(IV)-oxo oxidants affect the oxygen atom transfer ability? A computational study. Chem. Eur. J..

[75] Bordwell F.G., Cheng J.-P. (1991). Substituent effects on the stabilities of phenoxyl radicals and the acidities of phenoxyl radical cations. J. Am. Chem. Soc..

[76] Mayer J.M. (1998). Hydrogen atom abstraction by metal-oxo complexes: Understanding the analogy with organic radical reactions. Acc. Chem. Res..

[77] Barman P., Upadhyay P., Faponle A.S., Kumar J., Nag S.S., Kumar D., Sastri C.V., de Visser S.P. (2016). Deformylation reaction by a nonheme manganese(III)-peroxo complex via initial hydrogen atom abstraction. Angew. Chem. Int. Ed..

[78] Cantú Reinhard F.G., Barman P., Mukherjee G., Kumar J., Kumar D., Kumar D., Sastri C.V., de Visser S.P. (2017). Keto-enol tautomerization triggers an electrophilic aldehyde deformylation reaction by a nonheme manganese(III)-peroxo complex. J. Am. Chem. Soc..

[79] Latifi R., Valentine J.S., Nam W., de Visser S.P. (2012). Predictive studies of H-atom abstraction reactions by an iron(IV)-oxo corrole cation radical oxidant. Chem. Commun..

[80] Kumar D., Sastry G.N., de Visser S.P. (2012). Axial ligand effect on the rate constant of aromatic hydroxylation by iron(IV)-oxo complexes mimicking cytochrome P450 enzymes. J. Phys. Chem. B.

[81] Kumar D., Sastry G.N., de Visser S.P. (2011). Effect of the axial ligand on substrate sulfoxidation mediated by iron(IV)-oxo porphyrin cation radical oxidants. Chem. Eur. J..

[82] Kumar D., Karamzadeh B., Sastry G.N., de Visser S.P. (2010). What factors influence the rate constant of substrate epoxidation by Compound I of cytochrome P450 and analogous iron(IV)-oxo oxidants. J. Am. Chem. Soc..

[83] Hernández-Ortega A., Quesne M.G., Bui S., Heuts D.P.H.M., Steiner R.A., Heyes D.J., de Visser S.P., Scrutton N.S. (2014). Origin of the proton-transfer step in the cofactor-free 1-H-3-hydroxy-4-oxoquinaldine 2,4-dioxygenase: Effect of the basicity of an active site His residue. J. Biol. Chem..

[84] Hernández-Ortega A., Quesne M.G., Bui S., Heyes D.J., Steiner R.A., Scrutton N.S., de Visser S.P. (2015). Catalytic mechanism of cofactor-free dioxygenases and how they circumvent spin-forbidden oxygenation of their substrates. J. Am. Chem. Soc..

[85] Faponle A.S., Quesne M.G., de Visser S.P. (2016). Origin of the regioselective fatty-acid hydroxylation versus decarboxylation by a cytochrome P450 peroxygenase: What drives the reaction to biofuel production?. Chem. Eur. J..

[86] Fellner M., Siakkou E., Faponle A.S., Tchesnokov E.P., de Visser S.P., Wilbanks S.M., Jameson G.N.L. (2016). Influence of cysteine 164 on active site structure in rat cysteine dioxygenase. J. Biol. Inorg. Chem..

[87] Dakhili S.Y.T., Caslin S.A., Faponle A.S., Quayle P., de Visser S.P., Wong L.S. (2017). Recombinant silicateins as model biocatalysts in organosiloxane chemistry. Proc. Natl. Acad. Sci. USA.

[88] Cantú Reinhard F.G., de Visser S.P. (2017). Biodegradation of cosmetics products: A computational study of Cytochrome P450 metabolism of phthalates. Inorganics.

[89] Godfrey E., Porro C.S., de Visser S.P. (2008). Comparative quantum mechanics/molecular mechanics (QM/MM) and density functional theory calculations on the oxo-iron species of taurine/α-ketoglutarate dioxygenase. J. Phys. Chem. A.

[90] Quesne M.G., Latifi R., Gonzalez-Ovalle L.E., Kumar D., de Visser S.P. (2014). Quantum mechanics/molecular mechanics study on the oxygen binding and substrate hydroxylation step in AlkB repair enzymes. Chem. Eur. J..

[91] Kumar D., Thiel W., de Visser S.P. (2011). Theoretical study on the mechanism of the oxygen activation process in cysteine dioxygenase enzymes. J. Am. Chem. Soc..

[92] Prokop K.A., de Visser S.P., Goldberg D.P. (2010). Unprecedented rate enhancements of hydrogen-atom transfer to a manganese(V)-oxo corrolazine complex. Angew. Chem. Int. Ed..

[93] Vardhaman A.K., Sastri C.V., Kumar D., de Visser S.P. (2011). Nonheme ferric hydroperoxo intermediates are efficient oxidants of bromide oxidation. Chem. Commun..

[94] Vardhaman A.K., Barman P., Kumar S., Sastri C.V., Kumar D., de Visser S.P. (2013). Comparison of the reactivity of nonheme iron(IV)-oxo versus iron(IV)-imido complexes: Which is the better oxidant?. Angew. Chem. Int. Ed..

[95] Kumar S., Faponle A.S., Barman P., Vardhaman A.K., Sastri C.V., Kumar D., de Visser S.P. (2014). Long-range electron transfer triggers mechanistic differences between iron(IV)-oxo and iron(IV)-imido oxidants. J. Am. Chem. Soc..

[96] Sainna M.A., Kumar S., Kumar D., Fornarini S., Crestoni M.E., de Visser S.P. (2015). A comprehensive test set of epoxidation rate constants by iron(IV)-oxo porphyrin complexes. Chem. Sci..

[97] Yang T., Quesne M.G., Neu H.M., Cantú Reinhard F.G., Goldberg D.P., de Visser S.P. (2016). Singlet versus triplet reactivity in an Mn(V)-Oxo species: Testing theoretical predictions against experimental evidence. J. Am. Chem. Soc..

[98] Barman P., Faponle A.S., Vardhaman A.K., Angelone D., Löhr A.-M., Browne W.R., Comba P., Sastri C.V., de Visser S.P. (2016). Influence of ligand architecture in tuning reaction bifurcation pathways for chlorite oxidation by nonheme iron complexes. Inorg. Chem..

[99] Cantú Reinhard F.G., Sainna M.A., Upadhyay P., Balan G.A., Kumar D., Fornarini S., Crestoni M.E., de Visser S.P. (2016). A systematic account on aromatic hydroxylation by a cytochrome P450 model Compound I: A low-pressure mass spectrometry and computational study. Chem. Eur. J..

[100] Cantú Reinhard F.G., Faponle A.S., de Visser S.P. (2016). Substrate sulfoxidation by an iron(IV)-oxo complex: Benchmarking computationally calculated barrier heights to experiment. J. Phys. Chem. A.

[101] Cantú Reinhard F.G., Fornarini S., Crestoni M.E., de Visser S.P. (2018). Hydrogen atom versus hydride transfer in cytochrome P450 oxidations: A combined mass spectrometry and computational study. Eur. J. Inorg. Chem..

[102] Fowler N.J., Blanford C.F., Warwicker J., de Visser S.P. (2017). Prediction of reduction potentials of copper proteins with continuum electrostatics and density functional theory. Chem. Eur. J..

[103] Kaczmarek K.A., Malhotra A., Balan G.A., Timmins A., de Visser S.P. (2018). Nitrogen reduction to ammonia on a biomimetic mononuclear iron center: Insights into the nitrogenase enzyme. Chem. Eur. J..

[104] Frisch M.J., Trucks G.W., Schlegel H.B., Scuseria G.E., Robb M.A., Cheeseman J.R., Scalmani G., Barone V., Petersson G.A., Nakatsuji H. (2016). Gaussian 09, Revision A.02.

[105] Becke A.D. (1993). Density-functional thermochemistry. III. The role of exact exchange. J. Chem. Phys..

[106] Lee C., Yang W., Parr R.G. (1988). Development of the Colle-Salvetti correlation-energy formula into a functional of the electron density. Phys. Rev. B.

[107] Hay P.J., Wadt W.R. (1985). Ab initio effective core potentials for molecular calculations. Potentials for the transition metal atoms Sc to Hg. J. Chem. Phys..

[108] Sainna M.A., Sil D., Sahoo D., Martin B., Rath S.P., Comba P., de Visser S.P. (2015). Spin state ordering in hydroxo-bridged diiron(III)bisporphyrin complexes. Inorg. Chem..

[109] De Visser S.P. (2005). What affects the quartet-doublet energy splitting in peroxidase enzymes?. J. Phys. Chem. A.

[110] De Visser S.P., Ogliaro F., Harris N., Shaik S. (2001). Multi-state epoxidation of ethene by cytochrome P450: A quantum chemical study. J. Am. Chem. Soc..

[111] De Visser S.P., Shaik S., Sharma P.K., Kumar D., Thiel W. (2003). Active species of horseradish peroxidase (HRP) and cytochrome P450: Two electronic chameleons. J. Am. Chem. Soc..

[112] Fisher M.T., Sligar S.G. (1985). Control of heme protein redox potential and reduction rate: Linear free energy relation between potential and ferric spin state equilibrium. J. Am. Chem. Soc..

[113] Ogliaro F., Cohen S., de Visser S.P., Shaik S. (2000). Medium polarization and hydrogen bonding effects on Compound I of cytochrome P450: What kind of a radical is it really?. J. Am. Chem. Soc..

[114] Ogliaro F., de Visser S.P., Cohen S., Kaneti J., Shaik S. (2001). The experimentally elusive oxidant of cytochrome P450: A theoretical “trapping” defining more closely the “real” species. ChemBioChem.

[115] Dolinsky T.J., Czodrowski P., Li H., Nielsen J.E., Jensen J.H., Klebe G., Baker N.A. (2007). PDB2PQR: Expanding and upgrading automated preparation of biomolecular structures for molecular simulations. Nucl. Acids Res..

[116] Brooks B.R., Bruccoleri R.E., Olafson B.D., States D.J., Swaminathan S., Karplus M. (1983). CHARMM: A program for macromolecular energy, minimization, and dynamics calculations. J. Comput. Chem..

[117] Schafer A., Horn H., Ahlrichs R. (1992). Fully optimized contracted Gaussian basis sets for atoms Li to Kr. J. Chem. Phys..

[118] Sherwood P., de Vries A.H., Guest M.F., Schreckenbach G., Catlow C.R.A., French S.A., Sokol A.A., Bromley S.T., Thiel W., Turner A.J. (2003). QUASI: A general purpose implementation of the QM/MM approach and its application to problems in catalysis. J. Mol. Struct. Theochem..

[119] Smith W., Forester T.R. (1996). DL_POLY_2.0: A general-purpose parallel molecular dynamics simulation package. J. Mol. Graph..

[120] Bakowies D., Thiel W. (1996). Hybrid models for combined quantum mechanical and molecular mechanical approaches. J. Phys. Chem..

[121] Hitzenberger M., Hofer T.S. (2015). Probing the range of applicability of structure- and energy-adjusted QM/MM link bonds. J. Comput. Chem..

